# Supplementary material for: Resolving the full spectrum of human genome variation using Linked-Reads
Source: Genome Res. 2019 Apr;29(4):635–45. doi: 10.1101/gr.234443.118 (PMC6442396; doi:10.1101/gr.234443.118)
Supplement: Supplemental Material [file supp_gr.234443.118_Supplemental_longranger-2.2.zip › longranger-2.2.tar.bz2!/lr2.2-public/lib/stan/cmdstan/stan/doc/api/html/agrad_2fwd_2matrix_2mdivide__left__tri__low_8hpp_source.html]

Stan: src/stan/agrad/fwd/matrix/mdivide\_left\_tri\_low.hpp Source File


|  |  |
| --- | --- |
|  | Stan  2.5.0  probability, sampling & optimization |


- Main Page
- Related Pages
- Namespaces
- Classes
- Files

- File List
- File Members

All Classes Namespaces Files Functions Variables Typedefs Enumerations Enumerator Friends Macros Pages

- src
- stan
- agrad
- fwd
- matrix

mdivide\_left\_tri\_low.hpp

Go to the documentation of this file.

1 #ifndef STAN\_\_AGRAD\_\_FWD\_\_MATRIX\_\_MDIVIDE\_LEFT\_TRI\_LOW\_HPP

2 #define STAN\_\_AGRAD\_\_FWD\_\_MATRIX\_\_MDIVIDE\_LEFT\_TRI\_LOW\_HPP

3

4 #include <vector>

5 #include <stan/math/matrix/Eigen.hpp>

6 #include <stan/math/matrix/typedefs.hpp>

7 #include <stan/math/matrix/mdivide\_left.hpp>

8 #include <stan/math/error\_handling/matrix/check\_multiplicable.hpp>

9 #include <stan/agrad/fwd/matrix/typedefs.hpp>

10 #include <stan/agrad/fwd/matrix/to\_fvar.hpp>

11 #include <stan/agrad/fwd/matrix/multiply.hpp>

12 #include <stan/agrad/fwd/matrix/inverse.hpp>

13 #include <stan/agrad/fwd/fvar.hpp>

14 #include <stan/math/error\_handling/matrix/check\_square.hpp>

15

16 namespace stan {

17  namespace agrad {

18

19  template<typename T,int R1, int C1, int R2, int C2>

20  inline

21  Eigen::Matrix<fvar<T>,R1,C1>

22  mdivide\_left\_tri\_low(const Eigen::Matrix<fvar<T>, R1, C1>& A,

23  const Eigen::Matrix<fvar<T>, R2, C2>& b) {

24  using stan::math::multiply;

25  using stan::math::mdivide\_left;

26  stan::math::check\_square("mdivide\_left\_tri\_low(%1%)",A,"A",(double\*)0);

27  stan::math::check\_multiplicable("mdivide\_left\_tri\_low(%1%)",A,"A",

28  b,"b",(double\*)0);

29

30  Eigen::Matrix<T,R1,C2> inv\_A\_mult\_b(A.rows(),b.cols());

31  Eigen::Matrix<T,R1,C2> inv\_A\_mult\_deriv\_b(A.rows(),b.cols());

32  Eigen::Matrix<T,R1,C1> inv\_A\_mult\_deriv\_A(A.rows(),A.cols());

33  Eigen::Matrix<T,R1,C1> val\_A(A.rows(),A.cols());

34  Eigen::Matrix<T,R1,C1> deriv\_A(A.rows(),A.cols());

35  Eigen::Matrix<T,R2,C2> val\_b(b.rows(),b.cols());

36  Eigen::Matrix<T,R2,C2> deriv\_b(b.rows(),b.cols());

37  val\_A.setZero();

38  deriv\_A.setZero();

39

40  for (size\_type j = 0; j < A.cols(); j++) {

41  for(size\_type i = j; i < A.rows(); i++) {

42  val\_A(i,j) = A(i,j).val\_;

43  deriv\_A(i,j) = A(i,j).d\_;

44  }

45  }

46

47  for (size\_type j = 0; j < b.cols(); j++) {

48  for(size\_type i = 0; i < b.rows(); i++) {

49  val\_b(i,j) = b(i,j).val\_;

50  deriv\_b(i,j) = b(i,j).d\_;

51  }

52  }

53

54  inv\_A\_mult\_b = mdivide\_left(val\_A, val\_b);

55  inv\_A\_mult\_deriv\_b = mdivide\_left(val\_A, deriv\_b);

56  inv\_A\_mult\_deriv\_A = mdivide\_left(val\_A, deriv\_A);

57

58  Eigen::Matrix<T,R1,C2> deriv(A.rows(), b.cols());

59  deriv = inv\_A\_mult\_deriv\_b - multiply(inv\_A\_mult\_deriv\_A, inv\_A\_mult\_b);

60

61  return stan::agrad::to\_fvar(inv\_A\_mult\_b, deriv);

62  }

63

64  template<typename T,int R1, int C1, int R2, int C2>

65  inline

66  Eigen::Matrix<fvar<T>,R1,C1>

67  mdivide\_left\_tri\_low(const Eigen::Matrix<double, R1, C1>& A,

68  const Eigen::Matrix<fvar<T>, R2, C2>& b) {

69  using stan::math::multiply;

70  using stan::math::mdivide\_left;

71  stan::math::check\_square("mdivide\_left\_tri\_low(%1%)",A,"A",(double\*)0);

72  stan::math::check\_multiplicable("mdivide\_left\_tri\_low(%1%)",A,"A",

73  b,"b",(double\*)0);

74

75  Eigen::Matrix<T,R1,C2> inv\_A\_mult\_b(A.rows(),b.cols());

76  Eigen::Matrix<T,R1,C2> inv\_A\_mult\_deriv\_b(A.rows(),b.cols());

77  Eigen::Matrix<T,R2,C2> val\_b(b.rows(),b.cols());

78  Eigen::Matrix<T,R2,C2> deriv\_b(b.rows(),b.cols());

79  Eigen::Matrix<double,R1,C1> val\_A(A.rows(),A.cols());

80  val\_A.setZero();

81

82  for (size\_type j = 0; j < A.cols(); j++) {

83  for(size\_type i = j; i < A.rows(); i++) {

84  val\_A(i,j) = A(i,j);

85  }

86  }

87

88  for (size\_type j = 0; j < b.cols(); j++) {

89  for(size\_type i = 0; i < b.rows(); i++) {

90  val\_b(i,j) = b(i,j).val\_;

91  deriv\_b(i,j) = b(i,j).d\_;

92  }

93  }

94

95  inv\_A\_mult\_b = mdivide\_left(val\_A, val\_b);

96  inv\_A\_mult\_deriv\_b = mdivide\_left(val\_A, deriv\_b);

97

98  Eigen::Matrix<T,R1,C2> deriv(A.rows(), b.cols());

99  deriv = inv\_A\_mult\_deriv\_b;

100

101  return stan::agrad::to\_fvar(inv\_A\_mult\_b, deriv);

102  }

103

104  template<typename T,int R1, int C1, int R2, int C2>

105  inline

106  Eigen::Matrix<fvar<T>,R1,C1>

107  mdivide\_left\_tri\_low(const Eigen::Matrix<fvar<T>, R1, C1>& A,

108  const Eigen::Matrix<double, R2, C2>& b) {

109  using stan::math::multiply;

110  using stan::math::mdivide\_left;

111  stan::math::check\_square("mdivide\_left\_tri\_low(%1%)",A,"A",(double\*)0);

112  stan::math::check\_multiplicable("mdivide\_left\_tri\_low(%1%)",A,"A",

113  b,"b",(double\*)0);

114

115  Eigen::Matrix<T,R1,C2> inv\_A\_mult\_b(A.rows(),b.cols());

116  Eigen::Matrix<T,R1,C1> inv\_A\_mult\_deriv\_A(A.rows(),A.cols());

117  Eigen::Matrix<T,R1,C1> val\_A(A.rows(),A.cols());

118  Eigen::Matrix<T,R1,C1> deriv\_A(A.rows(),A.cols());

119  val\_A.setZero();

120  deriv\_A.setZero();

121

122  for (size\_type j = 0; j < A.cols(); j++) {

123  for(size\_type i = j; i < A.rows(); i++) {

124  val\_A(i,j) = A(i,j).val\_;

125  deriv\_A(i,j) = A(i,j).d\_;

126  }

127  }

128

129  inv\_A\_mult\_b = mdivide\_left(val\_A, b);

130  inv\_A\_mult\_deriv\_A = mdivide\_left(val\_A, deriv\_A);

131

132  Eigen::Matrix<T,R1,C2> deriv(A.rows(), b.cols());

133  deriv = -multiply(inv\_A\_mult\_deriv\_A, inv\_A\_mult\_b);

134

135  return stan::agrad::to\_fvar(inv\_A\_mult\_b, deriv);

136  }

137  }

138 }

139 #endif

check\_multiplicable.hpp

inverse.hpp

typedefs.hpp

stan::agrad::mdivide\_left

Eigen::Matrix< fvar< T >, R1, C2 > mdivide\_left(const Eigen::Matrix< fvar< T >, R1, C1 > &A, const Eigen::Matrix< fvar< T >, R2, C2 > &b)

**Definition:** mdivide\_left.hpp:25

stan::agrad::fvar

**Definition:** fvar.hpp:13

stan::agrad::to\_fvar

fvar< T > to\_fvar(const T &x)

**Definition:** to\_fvar.hpp:17

typedefs.hpp

stan::agrad::multiply

Eigen::Matrix< fvar< T >, R1, C1 > multiply(const Eigen::Matrix< fvar< T >, R1, C1 > &m, const fvar< T > &c)

**Definition:** multiply.hpp:21

stan::math::check\_multiplicable

bool check\_multiplicable(const char \*function, const T1 &y1, const char \*name1, const T2 &y2, const char \*name2, T\_result \*result)

**Definition:** check\_multiplicable.hpp:15

stan::agrad::size\_type

Eigen::Matrix< double, Eigen::Dynamic, Eigen::Dynamic >::Index size\_type

**Definition:** typedefs.hpp:14

fvar.hpp

Eigen.hpp

to\_fvar.hpp

mdivide\_left.hpp

multiply.hpp

stan::math::multiply

boost::enable\_if\_c< boost::is\_arithmetic< T >::value, Eigen::Matrix< double, R, C > >::type multiply(const Eigen::Matrix< double, R, C > &m, T c)

Return specified matrix multiplied by specified scalar.

**Definition:** multiply.hpp:25

stan::agrad::mdivide\_left\_tri\_low

Eigen::Matrix< fvar< T >, R1, C1 > mdivide\_left\_tri\_low(const Eigen::Matrix< fvar< T >, R1, C1 > &A, const Eigen::Matrix< fvar< T >, R2, C2 > &b)

**Definition:** mdivide\_left\_tri\_low.hpp:22

stan::math::check\_square

bool check\_square(const char \*function, const Eigen::Matrix< T\_y, Eigen::Dynamic, Eigen::Dynamic > &y, const char \*name, T\_result \*result)

Return true if the specified matrix is square.

**Definition:** check\_square.hpp:24

stan::math::mdivide\_left

Eigen::Matrix< typename boost::math::tools::promote\_args< T1, T2 >::type, R1, C2 > mdivide\_left(const Eigen::Matrix< T1, R1, C1 > &A, const Eigen::Matrix< T2, R2, C2 > &b)

Returns the solution of the system Ax=b.

**Definition:** mdivide\_left.hpp:24

check\_square.hpp


---

[ Stan Home Page ]

*© 2011–2014,
Stan Development Team.*
